# Comparison of the Impact of VRP-034 and Polymyxin B upon Markers of Kidney Injury in Human Proximal Tubule Monolayers In Vitro

**DOI:** 10.3390/antibiotics13060530

**Published:** 2024-06-06

**Authors:** Keith Pye, Elena Tasinato, Siannah Shuttleworth, Claire Devlin, Colin Brown

**Affiliations:** Newcells Biotech Ltd., The Biosphere, Newcastle-upon-Tyne NE4 5BX, UK

**Keywords:** drug-induced kidney injury, polymyxin B, VRP-034, KIM-1, nephrotoxicity, cystatin-C, clusterin, biomarkers, aProximate

## Abstract

In this study, we assessed the impact of commercially available polymyxin B against VRP-034 (novel formulation of polymyxin B) using a validated in vitro human renal model, aProximate^TM^. Freshly isolated primary proximal tubule cells (PTCs) were cultured in Transwell plates and treated with various concentrations of the formulations for up to 48 h. The functional expression of megalin–cubilin receptors in PTC monolayers was validated using FITC-conjugated albumin uptake assays. Polymyxin B and VRP-034 were evaluated at six concentrations (0.3, 1, 3, 10, 30, and 60 µM), and nephrotoxicity was assessed through measurements of transepithelial electrical resistance (TEER), intracellular adenosine triphosphate (ATP) levels, lactate dehydrogenase (LDH) release, and novel injury biomarkers [kidney injury molecule-1 (KIM-1), neutrophil gelatinase-associated lipocalin (NGAL), and clusterin]. Additionally, histological analysis using annexin V apoptosis staining was performed. Our results indicated a significant decrease in TEER with polymyxin B at concentrations ≥10 μM compared to VRP-034. Toxic effects were observed from ATP and LDH release only at concentrations ≥30 μM for both formulations. Furthermore, injury biomarker release was higher with polymyxin B compared to VRP-034, particularly at concentrations ≥10 µM. Histologically, polymyxin B-treated PTCs showed increased apoptosis compared to VRP-034-treated cells. Overall, VRP-034 demonstrated improved tolerance in the aProximate^TM^ model compared to polymyxin B, suggesting its potential as a safer alternative for renal protection.

## 1. Introduction 

Antibiotic resistance has emerged as one of the most dangerous threats to human health over the past decade, with significant implications on the global healthcare sector in terms of patient care [1]. This has led to an urgent need for new or improved treatments to combat resistant bacterial pathogens [2]. Polymyxin B, a last-resort antibiotic, is effective against multidrug-resistant infections but its use is limited by significant nephrotoxicity [3]. Polymyxin B’s nephrotoxicity restricts its therapeutic window, with toxic effects occurring in up to 40% of patients and up to 60% when combined with other nephrotoxic agents [4,5]. This results in a narrow window being created, within which polymyxin B can be used to effectively fight bacterial infections whilst not inducing acute kidney injury in patients suffering from these. In fact, this is considered a key obstacle to overcome, with emphasis on the careful considerations clinicians should take prior to considering polymyxin B therapy, due to the maximum recommended dosing being 3 mg/kg/day [6]. This very narrow window highlights the major limitation of this as a therapy and, considering antimicrobial strains becoming increasingly prevalent, shows how essential alternatives to this therapy, or amelioration of the concentration at which nephrotoxic events occur, will be over the coming years. 

Although the precise mechanism of toxicity is unclear, the renal proximal tubule has been well-documented as the site of cellular damage, resulting in loss of kidney function and eventual failure if left unchecked. It is proposed that polymyxin B accumulates and interferes at the mitochondrial level of the tubular epithelium, resulting in the generation of reactive oxygen species, cellular injury, and apoptosis [7]. This accumulation occurs as a result of the efficient and extensive tubular reabsorption of polymyxin B from the lumen of the tubule back into the plasma via the proximal tubular epithelial cells, which results in a highly concentrated dose of this compound being exposed to the tubular epithelium throughout the duration of treatment [8]. Therefore, this requires development of reformulated variants of polymyxin B that attempt to address these safety concerns whilst retaining the efficacious bactericidal response. 

VRP-034 represents an innovative approach by introducing a supramolecular cationic formulation (SMC) that incorporates polymyxin B (as active compound), l-arginine, and low-molecular-weight dextran (LMWD). The formulation leverages electrostatic interactions within a meticulously determined charge-to-molecular weight ratio. Its primary aim is to preserve the inherent antimicrobial effectiveness of polymyxin B while addressing the challenge of polymyxin-B-induced nephrotoxicity. Achieving this balance can expand therapeutic options, allowing for safer dosing and broader patient use, which is crucial in combating antibiotic resistance. Traditionally, the nephrotoxicity of polymyxin B has necessitated suboptimal dosing to minimize kidney damage, which unfortunately can contribute to the development of drug resistance. Safer polymyxin B can enable healthcare providers to administer the full, effective doses required to thoroughly eradicate infections. This not only improves treatment outcomes but also reduces the resistance selection for polymyxin B, thereby extending the useful life of the drug. Previous studies have demonstrated VRP-034’s promising safety profile as compared to commercially available polymyxin B in animal models [9,10,11,12,13,14], thereby paving the way for further exploration, including a comprehensive safety evaluation in this study using human primary renal proximal tubule cells (PTCs) isolated from a transplant-grade kidney.

The renal PTC are well-documented to express a wide variety of transporters from the two transporter superfamilies, ATP-binding cassette (ABC) and solute carrier (SLC) transporters, within their apical and basolateral membranes, which play an important role in the secretion and reabsorption of a variety of endogenous compounds, as well as xenobiotics. Alongside these transporters of, typically, small molecules, there are also various transporters or transport mechanisms that have a propensity to transport larger molecules, which are often expressed at the apical membrane of cells and involved in the reabsorption of filtered proteins or larger molecules, such as exogenous polypeptides. One of the key transport mechanisms at the lumen of the proximal tubule is the megalin and cubilin large endocytic receptor complex. This receptor complex has been well-defined for its role in the accumulation of compounds resulting in nephrotoxicity, including polymyxin B [15,16]. Upon intracellular accumulation of polymyxin B, the PTCs are exposed to high concentrations of the antibiotic, leading to cell injury, apoptosis, and tubular damage. 

There has been growing momentum towards a paradigm shift from the classic measurements of kidney injury, such as serum creatinine and blood urea nitrogen, towards that of markers that are more specific to the kidney, and which can be detected in the blood or urine yielding a more sensitive and specific measure [17]. Due to the proximal tubule epithelium being the site of movement of many xenobiotics between the blood and the lumen, this cell type has been of particular interest in investigating the production and secretion of many of these biomarkers, including kidney injury molecule-1 (KIM-1), clusterin, and neutrophil gelatinase-associated lipocalin (NGAL). With this in mind, recent research has moved towards identifying an in vitro model of the proximal tubule that can be used as a predictive screening tool for compounds that may be exposed to the proximal tubule to determine their nephrotoxic potential prior to entering the clinic. A recent validation study investigating the use of freshly isolated primary human proximal tubule cells found the use of these biomarker combinations to be highly sensitive and specific [16]. One of these biomarkers, KIM-1, was also used in the investigation of VRP-034 in vivo to determine the potential of kidney toxicity against commercially available polymyxin B in rats, alongside classic measurements of kidney injury, such as BUN, serum creatinine, and histopathology [9]. The present study aimed to investigate whether the response seen in the pre-clinical animal model was comparable in a validated in vitro human model, aProximate [9,10,11,12,13]. This model utilises freshly isolated primary human PTCs, which express many relevant transporters of endogenous and exogenous substrates, allowing the recapitulation of the in vivo proximal tubule in the context of drug transport and nephrotoxicity. Megalin and cubilin were investigated at the functional level in this study by quantifying FITC-albumin uptake. Albumin is a substrate for both proteins of this complex and, thus, can be partially inhibited by receptor-associated protein (RAP), which binds specifically to megalin. This study included the incubation of FITC-albumin in the presence and absence of RAP to demonstrate the functional activity of the receptors through uptake and inhibition of the labelled albumin. 

Nephrotoxic potential of commercially available polymyxin B and VRP-034 were assessed by measuring three markers of gross viability [transepithelial electrical resistance (TEER), intracellular adenosine triphosphate (ATP), and lactate dehydrogenase (LDH) release], as well as quantifying the clinically relevant biomarkers KIM-1, NGAL, and clusterin. In addition to these functional readouts, annexin V staining was also carried out to provide visual representation of the effects of the formulation on the PTC monolayers.

## 2. Results

### 2.1. Intracellular Accumulation of Megalin/Cubilin-Dependent FITC-Albumin into hPTC Monolayers 

**Figure 1:**
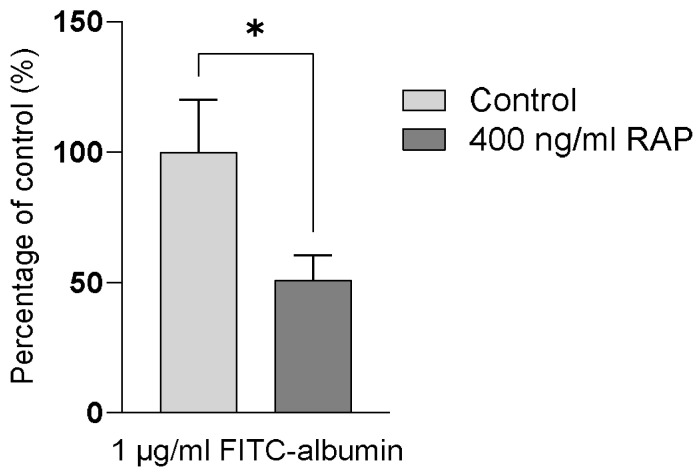
Intracellular uptake of FITC-albumin across the apical membrane of hPTCs in the absence (light grey bars) and presence (dark grey bars) of 400 ng/mL RAP, expressed as a percentage of the control monolayers. Data are from a representative donor derived from one human kidney. Data are presented as the mean of three replicates. Error bars capture SD. Significance is denoted by, * = *p* < 0.05.

Confluent PTC monolayers were assayed to determine functional expression of megalin and cubilin endocytic receptor complex by measuring FITC-albumin uptake across the apical membrane. This was performed in the presence and absence of an inhibitor of this complex, RAP, at 400 ng/mL. The representative data show a mean accumulation across the apical membrane of 156.4 ± 12.85 ng/mL FITC-albumin, shown as 100% of uptake in Figure 1. In the presence of RAP, there is a significant decrease in uptake of FITC-albumin across the apical membrane, from 156.4 ± 12.8 ng/mL to 79.6 ± 10.6 ng/mL (*p* < 0.05), shown as ~50% inhibition in Figure 1. 

### 2.2. Comparison of VRP-034 and Polymyxin B Treatment on Gross Viability Markers from hPTC Monolayers 

**Figure 2:**
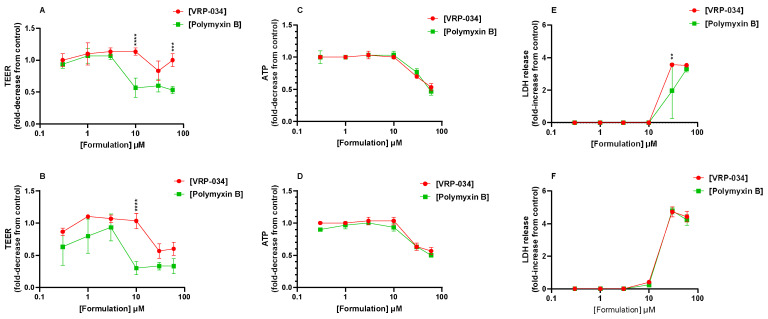
Viability markers measured in hPTC monolayers derived from human kidney, presented as the fold-change from control monolayers. TEER measurements after 24 (**A**) and 48 h (**B**), LDH-release after 24 (**C**) and 48 h (**D**), and intracellular ATP after 24 (**E**) and 48 h (**F**). Green points pertain to marketed polymyxin B, whilst red points denote the VRP-034 novel formulation. Each data point represents the mean of three technical replicates. Error bars capture SD. Significance is denoted by ** = *p* < 0.01, *** = *p* < 0.001, **** = *p* < 0.0001.

Confluent human PTC monolayers were treated with a range of concentrations of polymyxin B or VRP-034 (0.3–60 µM) for either 24 or 48 h. Measurements of barrier integrity, cell health, and cell death were all taken and are displayed in Figure 2 as the fold-change from control monolayers, either as an increase (LDH) or decrease (TEER and ATP).

The TEER data show a similar level of resistance after treatment with both compounds up until 10 µM, at both 24 (A) and 48 h (B) of treatment. At 10 µM VRP-034 treatment, the TEER is similar to untreated control monolayers, whilst there is a ~50% decrease after 24 h polymyxin B treatment (*p* < 0.0001) and a 70% decrease after 48 h treatment (*p* < 0.0001). With an increase in VRP-034 concentration, there is a decrease in TEER by ~50% after 48 h. However, after a 24 h treatment, the monolayers appear to be less affected by increasing VRP-034 concentrations, with the TEER at 60 µM still significantly higher than that of 60 µM polymyxin B (*p* < 0.001). Intracellular ATP and LDH release exhibit similar patterns for both compounds until concentrations of ≥30 μM are reached. Although LDH levels appear notably higher after 30 µM VRP-034 treatment compared to polymyxin B. It is observed that there is considerable error in the 30 µM polymyxin B condition.

### 2.3. Comparison of VRP-034 and Polymyxin B Treatment on Biomarker Production from hPTC Monolayers 

**Figure 3:**
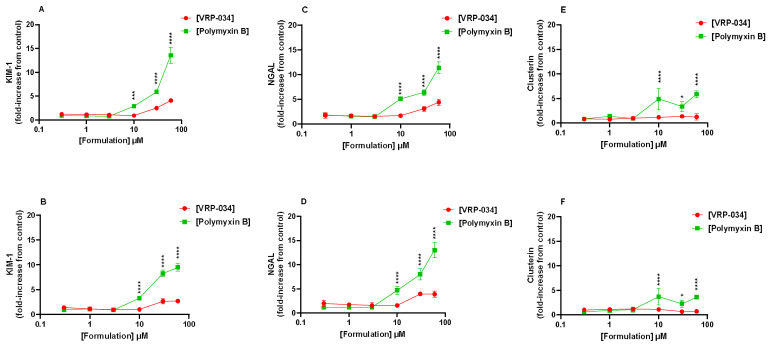
Biomarker secretion from hPTC monolayers, presented as the fold-increase from control monolayers. KIM-1 production after 24 (**A**) and 48 h (**B**), NGAL production after 24 (**C**) and 48 h (**D**), and clusterin production after 24 (**E**) and 48 h (**F**). Green points pertain to marketed polymyxin B whilst red points denote the VRP-034 novel formulation. Each data point is the mean of three technical replicates, except NGAL after 48 h 60 µM polymyxin B treatment, which is two replicates, one being excluded due to an aberrantly low data point, which was an outlier within the set of triplicates compared with the other 5 readouts from that same well. Error bars capture SD. Significance is denoted by, * = *p* < 0.05, *** = *p* < 0.001, **** = *p* < 0.0001.

Three biomarker proteins were measured at two different timepoints, of 24 and 48 h, presented in Figure 3. After 24 h of treatment with both formulations, KIM-1 is observed to increase but is up to ~three-fold greater in conditions with the standard polymyxin B than VRP-034 formulation from 10 µM and higher (*p* < 0.001). Interestingly this does not deviate after a further 24 h of treatment and there is even a slight reduction of KIM-1 secretion observed in conditions treated with both formulations of polymyxin B (Figure 3A,B). NGAL secretion follows a very similar profile, with a significantly greater level of the protein produced when exposed to the standard polymyxin B for 24 and 48 h of treatment from 10 µM (*p* < 0.0001). In clusterin, a significantly greater amount of protein is observed at both timepoints when exposed to 10 µM of marketed polymyxin B but not VRP-034 (*p* < 0.0001). This seems to then plateau, however, as there is no significant further increase at either timepoint upon increasing concentration.

### 2.4. Comparison of Effects of VRP-034 and Polymyxin B Treatment on Apoptosis (Annexin V-FITC) and Necrosis (PI) in hPTC Monolayers

Confluent human hPTC monolayers were treated with 50 µM cisplatin or a range of concentrations of polymyxin B or VRP-034. Annexin V-FITC Apoptosis Staining/Detection Kit (Abcam) was used to detect apoptotic and necrotic cells among treated and untreated monolayers via fluorescence microscopy. 

**Figure 4:**
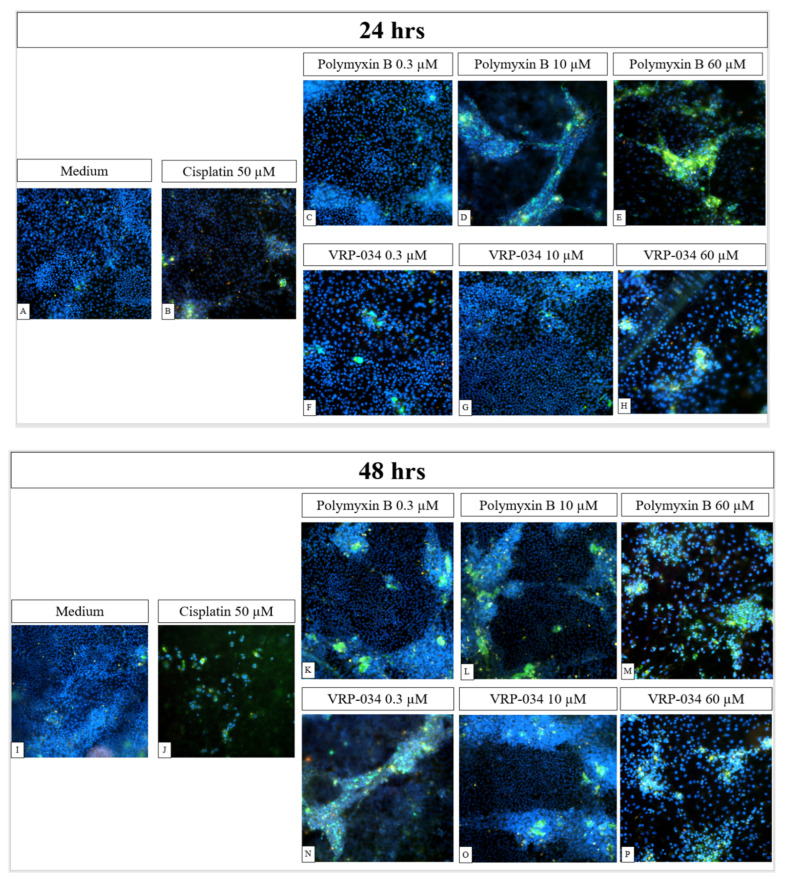
Fluorescent images of annexin V-FITC/PI-stained treated or untreated epithelial monolayers after 24 and 48 h of treatment. Cell nuclei are depicted in blue, while apoptotic cells are shown in green, and necrotic cells are shown in orange. After 24 h of treatment, polymyxin-B-treated cells (**C**–**E**) show increased positive annexin V-FITC staining as compared to equal concentrations of VRP-034 treatment (**F**–**H**). After 48 h of treatment, cells treated with polymyxin B (**K**–**M**) exert migration in large nodules, which show more intense annexin V staining as compared to VRP-034-treated cells (**N**–**P**). (**A**,**I**) medium only; (**B**,**J**) cisplatin; (**C**,**K**) 0.3 μM polymyxin B; (**D**,**L**) 10 μM polymyxin B; (**E**,**M**) 60 μM polymyxin B; (**F**,**N**) 0.3 μM VRP-034; (**G**,**O**)10 μM VRP-034; (**H**,**P**) 60 μM VRP-034.

**Figure 5:**
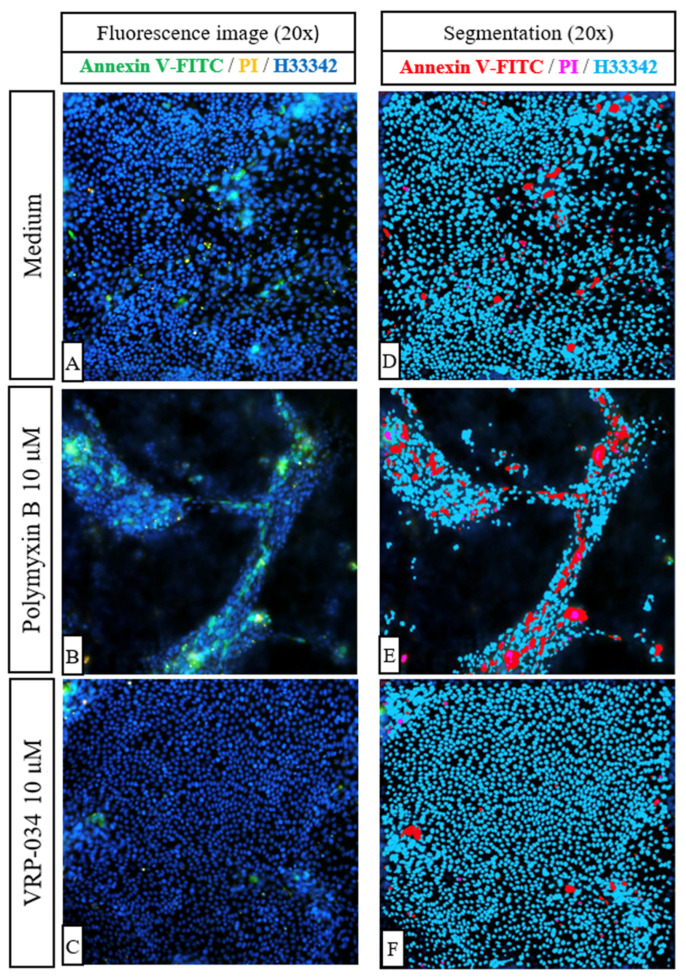
Examples of fluorescent images (**A**–**C**) of annexin V-FITC/PI-stained treated or untreated hPTCs monolayers and images of applied segmentation (**D**–**F**). After segmentation is applied to the images, nuclei are depicted in light blue, apoptotic cells are in red, and necrotic cells in pink.

After 24 h, some apoptotic and necrotic cells are visible in cisplatin-treated monolayers (Figure 4B). Cells treated with 0.3 µM polymyxin B (Figure 4C) migrate into nodules that stain positively for annexin V-FITC (Figure 4F). Furthermore, apoptotic nodule formation is noticeable at 10 µM and 60 µM polymyxin B concentrations (Figure 4D,E), the latter concentration leading to prominent apoptosis of the epithelium (Figure 4E). Interestingly, in the presence of 10 µM and 60 µM VRP-034 (Figure 4G,H), cells migrate into smaller nodules as compared to polymyxin B treatment and monolayers appear to be more uniform. After 48 h, extensive nodule formation is visible in hPTC monolayers treated with 0.3 µM polymyxin B, with several apoptotic cells present (Figure 4K). The same feature is displayed by cells treated with 0.3 µM VRP-034, with apoptotic cells appearing to be sparser across the nodule (Figure 4N). The number of apoptotic cells and nodules is noticeably higher when cells are treated with 10 μM polymyxin B (4 L) as compared to the same concentration of VRP-034 (4 O). More apoptotic cells are also present with 60 μM polymyxin B (4 M) as compared to the same concentration of VRP-034 (P). Moreover, the applied segmentation of fluorescence images shows that after just 24 h of treatment with 10 µM of polymyxin B (Figure 5B,E), more apoptotic and necrotic cells are visible as compared to an equal concentration of VRP-034 (Figure 5C,F). 

## 3. Discussion

The dose-limiting toxicity aspect of polymyxin B is the key reason that there is reluctance to use this compound (and compounds within the same class), even in the face of increasing antibiotic resistance. This is due to the extensive tubular reabsorption and intracellular accumulation of polymyxin B, likely via the megalin/cubilin receptor to which it binds. Downstream toxicity events have yet to be fully understood but involves the induction of apoptosis, in part due to localisation to the mitochondria, where it may exert its cellular injury via free radical generation and oxidative stress [7,18]. We demonstrate here functional evidence of the megalin/cubilin complex by quantifying FITC-albumin uptake across the apical membrane of the PTCs and the resulting inhibition using a competitive inhibitor of megalin binding, RAP (Figure 1). Recently, it has been postulated that there are multiple kinetic profiles of megalin and cubilin for albumin [19]. This suggests that there are up to three mechanisms for albumin tubular reabsorption, which may become more relevant under increasing luminal concentrations of albumin, allowing sufficient recovery of albumin under nephrotic conditions. The reason for this incomplete inhibition is likely due to the complexity of multiple binding sites and differing binding affinities between megalin and cubilin substrates. It is, therefore, possible that, in the presence of RAP, there are still alternative binding sites for albumin and, therefore, a route into the cell, albeit in a lower quantity, which may explain why the inhibitory effects of RAP alone are not enough to fully block albumin uptake. 

A solution to polymyxin-B-related nephrotoxicity could be to reformulate the compound to limit the tubular uptake and, therefore, subsequent cellular injury. To measure such effects, suitable models must not only demonstrate functional uptake via relevant transport mechanisms, as we have shown, but must also respond accordingly at clinically relevant concentrations. Responses such as kidney injury biomarkers (e.g., KIM-, clusterin, NGAL, amongst others), which can be measured in certain in vitro systems, such as the one described in this study, have been shown previously to correlate with in vivo measurements. KIM-1, NGAL, and clusterin, in particular, have shown urinary output in both in vivo and in vitro models of acute kidney injury (AKI) in response to nephrotoxic compounds, such as cisplatin [20,21,22]. KIM-1 has been investigated in previous in vitro human models of tubular injury induced by polymyxin B, including an organ-on-a-chip model, which showed KIM-1 induction after treatment, comparative to structural analogues of polymyxin B [22]. One such reformulation of polymyxin B, VRP-034, has previously shown promising results in in vivo studies in rodent models, showing very little increase from the baseline of four key markers of kidney injury: KIM-1, cystatin-C, urea, and creatinine [9,12,14]. In contrast, marketed polymyxin B showed a significant increase in all four markers, with a ~three-fold increase in KIM-1 levels from the baseline [9]. This has been more recently expanded, again in rats, with three concentrations of both formulations measured, which, interestingly, showed that as concentration increased, the level of these markers produced by the VRP-034 were lower than those produced by an equivalent dose of the marketed formulation [12,13,14]. In the present study, after treatment with another substrate of megalin, polymyxin B, for 24 and 48 h, dose-dependent deleterious effects on all six readouts of nephrotoxicity were observed. Interestingly, of the gross viability markers, only TEER suggested an ameliorated response after VRP-034 treatment when compared to marketed polymyxin B (Figure 2). Additionally, this was most pronounced at both timepoints after 10 µM treatment with both compounds. Neither ATP nor LDH seemed affected after treatment with 10 µM at either timepoint, but upon increasing the dose (30 µM), the readouts were very similar for both compounds. Our biomarker data, however, show a significantly greater increase in each protein secreted after polymyxin B treatment compared with VRP-034 from 10 µM of each compound (Figure 3). This concentration is particularly interesting due to the reported clinical concentration range within blood plasma, ranging from 2–14 µg/mL [23]. With 10 µM polymyxin B in this study equating to ~13.85 µg/mL, we have observed that this is a concentration that is dangerously close to inducing tubular injury. This adds strength to the potential utility of VRP-034 because the data suggest this may be better tolerated in vivo at the same concentration or higher and is less restricted by the narrow therapeutic index that polymyxin B toleration forces upon clinicians. The fact that this is initiated prior to the onset of ATP depletion or LDH release (10 µM) shows the relevance of using these three proteins as biomarkers, as it suggests the cells have become stressed and begun to shed or secrete these proteins as part of that response. It is interesting that there is still a response from VRP-034, suggesting that there is still uptake into these cells, which ties in with data previously presented showing retention of VRP-034 within kidney tissue of rats, although to a significantly lesser extent than standard polymyxin B [11]. It could be that the tissue retention is within the proximal tubule, although further work should be carried out to determine this. Additionally, the fluorescence images (Figure 4 and Figure 5) demonstrate that polymyxin B treatment leads to more visible hPTC migration into apoptotic nodules and monolayer disruption as compared to VRP-034. Increased proliferation, dedifferentiation, and migration of renal tubular epithelial cells in vivo is associated with AKI [24], a syndrome which is notably associated with polymyxins [25]. Our data demonstrate that VRP-034 is less likely to induce hPTC migration into apoptotic nodules in vitro. To add strength to the current investigation and what we were limited by, is the ability to directly measure cellular uptake of both polymyxin B and the reformulated version, VRP-034. While we see an attenuated response from VRP-034 incubation and can infer that it is likely due to reduced uptake via megalin/cubilin, supported by the evidence of functional inhibition, we cannot rule out the possibility of an alternative mechanism occurring intracellularly. By tagging of these compounds with radio- or fluorescent molecules, physical uptake of these molecules into the primary PTCs would demonstrate whether the reduced sensitivity to VRP-034 was due to less intracellular uptake, giving important insight into the mechanistic understanding of the reduced sensitivity. In this instance, we could also demonstrate inhibition by co-treating in the presence or RAP or other inhibitors of the complex, to also better understand the role of the megalin/cubilin complex in the uptake of both of these molecules. 

## 4. Materials and Methods

### 4.1. Chemicals and Reagents

Therapeutic agent VRP-034 was supplied by Venus Medicine Research Centre, Baddi, India, and standard polymyxin B was manufactured by Bharat Serum and Vaccines, Mumbai, India, and were both reconstituted in water to a concentration of 10 mg/mL. The ELISA kits to quantify the amount of KIM-1, NGAL, and clusterin were bought from R&D Systems, Minneapolis, MN, USA. The Annexin V Apoptosis detection kit was purchased from Abcam (ab14085, Boston, MA, USA). Cis-diamineplatinum(II) di-chloride (cisplatin) was purchased from Sigma-Aldrich, Gillingham, UK. ATP kits were bought from Promega Corporation, Hampshire, UK, and LDH assay kits were bought from ThermoFisher Scientific, Waltham, MA, USA. Unless otherwise stated, all other reagents were sourced from Sigma-Aldrich, UK, and were of the highest purity grade available. 

For nephrotoxicity endpoints, human PTC monolayers were exposed to polymyxin B and VRP-034 at six concentrations (0.3, 1, 3, 10, 30, and 60 µM) for 24 and 48 h to both the apical and basolateral side of the cell layer. Notably, VRP-034 contains an equivalent amount of the active compound, polymyxin B, so the concentrations for VRP-034 are expressed as polymyxin B-equivalent concentrations. For example, 1 µM of VRP-034 refers to 1 µM of polymyxin B. Previous studies have shown VRP-034 to be equipotent to polymyxin B at similar concentrations [9,13]. The concentration of 10 µM polymyxin B corresponds to approximately ~13.85 µg/mL in humans. However, a wider range (>10 µM) of concentrations was examined to ensure physiological relevance. Cisplatin-treated cells were assayed as a positive control of nephrotoxicity and used at a concentration of 50 µM. In addition, a negative control containing medium only was included in parallel. Each condition was performed in triplicate.

### 4.2. Preparation of Primary Human Proximal Tubule Monolayers

Primary human PTCs used in this study were isolated from a transplant-grade kidney. The protocol for PTC isolation was adapted from a previous protocol [26] and is described briefly here. All cell culture work was performed in a class II vertical laminar flow hood to ensure sterility. The kidney was decapsulated and cortex slices taken, which were then minced to approximately 1 mm^3^ pieces before 50 mL of isolation medium were used to suspend every 5 g of minced tissue. Type 2 collagenase (activity of ≈300 units/mg, working concentration of 1 μg/mL) was added to the suspension to initiate the digestion of the tissue. The suspension was kept shaken for 1.5 to 2 h at 37 °C before cell separation. 

To separate the cells, the suspension was passed through a 40 μm nylon sieve to remove undigested material and then centrifuged for 7 min at 1600 rpm. In this and all subsequent centrifugation steps, the temperature was maintained at 4 °C. The resulting cell pellet was resuspended in fresh isolation medium before the cells were pelleted again by centrifugation at 1600 rpm for 7 min (this is considered the wash step). The cell pellet was then loosened and gently resuspended again in fresh isolation medium. 

To separate out the proximal tubule cells, the cell suspension was loaded on top of discontinuous Percoll gradients with densities of 1.04 g/mL and 1.07 g/mL and centrifuged at 3000 rpm for 25 min. After centrifugation, PTCs at the intersection of the gradients were aspirated and washed as previously described. The cells were resuspended in warm human renal epithelial growth medium (REGM). The cell yield was estimated using a haemocytometer after carefully resuspending the cell pellet in 1–2 mL REGM using a P1000 pipette (Gilson, Dunstable, UK). 

Isolated cells were seeded on to 96-well Transwell inserts (surface area = 0.143 cm^2^) at a density of 37,500 cells per insert to investigate nephrotoxicity endpoints. For generation of cell monolayers to study the potential mechanism of action of both compounds and cisplatin on the human PTCs, cells were seeded onto 24-well transparent ThinCert inserts (surface area = 0.33 cm^2^) at a density of 75,000 cells per insert. PTCs were also seeded on to T25 cell culture flasks at a density of 1.875 million cells. The medium was refreshed after 24 h of initial seeding, and also at day 3 and 5 of culture. PTCs were maintained in a humidified incubator at 37 °C with 95:5 air:CO_2_. Confluency of the monolayers was determined by visual inspection of the cell culture flask under a phase contrast microscope. Transepithelial electrical resistance (TEER) was used as an indicator of monolayer confluency on the Transwell inserts. The monolayer resistance was measured using an electric volt-ohmmeter (EVOM, World Precision Instruments, Hitchin, UK). The TEER of the monolayer, with the unit of Ω·cm^2^, was calculated by subtracting the base resistance created by the filter submerged in culture medium (160 Ω for 96-well Transwell inserts, 90 Ω for 24-well Transwell inserts) and then multiplying it by the surface area (0.143 cm^2^ for 96-well Transwell inserts, 0.33 cm^2^ for 24-well Transwell inserts) of the filter. Monolayers that demonstrated TEER in the range of 60–120 Ω·cm^2^ were used in experiments.

### 4.3. FITC-Albumin Uptake

Once cell monolayers reached confluence and a TEER of at least 60 Ω·cm^2^, the monolayers were washed 3× in warm Krebs buffer. The cells were then left to preincubate for ~30 min in warm Krebs buffer, before the FITC-albumin in Krebs was added to either the insert (apical chamber) or well (basolateral chamber). FITC-albumin was dosed at a concentration of 1 μg/mL for a period of 120 min in the presence and absence of RAP at 400 ng/mL, after which the monolayers were washed thrice with ice-cold Krebs to stop the reaction. The monolayers were then lysed in 50 µL SDS (0.1%) for 5 min and then gently scraped off the membranes before being transferred to a clear-bottomed 96-well plate for measurement of absorbance. The 1 μg/mL FITC-albumin solution was serially diluted in a two-fold manner to provide a standard curve against which the concentration of FITC-albumin could be calculated from.

### 4.4. TEER, ATP, and LDH Assays

TEER values were measured after the 24- and 48-h treatment timepoints. Each monolayer in both the 24-well ThinCert plates and the 96-well Transwells were collected using a volt-ohmmeter, EVOM^2^ from WPI, Sarasota, FL, USA.

Cell viability assays based on ATP production and LDH release were performed on test compound-treated monolayers performed in 96-well Transwell inserts after 24 and 48 h of treatment. Assay kits were used, and the manufacturer’s instructions were followed. The assay protocols are described briefly here: 

For the LDH assay, 50 µL of medium from the apical side of each well of the treatment plates was transferred into clean 96-well plates and equal volume of LDH reaction mixture was added. The samples were incubated at room temperature for 30 min before the reaction was stopped by adding 50 µL of stop solution. The absorbance was then taken at 490 nm and 680 nm using a microplate reader (BMG Labtech, Ortenberg, Germany). In addition to the amount of LDH released by the treated monolayers, total amount of LDH constitutively produced was also determined by lysing three control monolayers prior to medium sampling. This was carried out by adding 22 µL of 10× lysis buffer (included in the kit) into the cell culture medium (200 µL volume) for 30–40 min prior to running the LDH assay. 

For the ATP assay, an equal volume of CellTiter-Glo reagent was added to the wells of the 96-well Transwell plates after sampling for the assorted assays. Because the CellTiter-Glo reagent contained a lysis agent, luminescence reading was only taken after at least 15 min of incubation at room temperature. Luminescence reading was performed using a microplate reader (BMG Labtech, Germany). The linear relationship between ATP and cell number allows the bioluminescent signal generated by the untreated control monolayers to be representative of ‘100%’ cell viability. All other values are then expressed as a percentage of the signal of the control monolayers.

### 4.5. Biomarker Quantification

Singleplex ELISA plates from R&D Systems were used to quantify the amount of KIM-1, NGAL, and clusterin produced by the treated monolayers after 24 and 48 h of treatment. The manufacturer’s instructions were followed in running the assay. All samples were diluted separately for their optimal analysis: clusterin was diluted 1:60, NGAL and KIM-1 were diluted 1:10 with the provided reagent diluent before use in the assay. Each sample was in triplicate in the assay, so one sample/well was used in the ELISA quantification.

### 4.6. Annexin V-FITC/PI Apoptosis/Necrosis Assay Imaging 

After the period of treatment (24 or 48 h) the medium plus treatments were aspirated from the wells and then the Annexin V-FITC Apoptosis Staining/Detection Kit (ab14085, Abcam) protocol was followed according to manufacturer’s instructions with the addition of 10 μg/mL Hoechst 3342 staining dye solution as nuclear counterstain after fixation. The membranes were excised from the individual inserts using a scalpel (product number: INS4767) and then mounted on slides with coverslips. The fluorescence imaging was undertaken on the AxioImager, Zeiss International, Aalen, Germany. Three images per condition (concentration/time point) were taken at 20× magnification. Exposure time, light intensity, and focus offset were determined for each channel at the beginning of the imaging session and were consistently applied to all images. The assessment of apoptotic and necrotic cells per field was conducted via segmentation performed by Zeiss ZEN Blue Image Analysis. 

### 4.7. Calculations, Data Processing, and Statistics

#### Biomarker Calculations

Each biomarker was calculated as a concentration as determined from the standard curve (pg/mL). If the TEER of a particular monolayer dropped below the resistance of a typical monolayer (60 Ω·cm^2^), the analyte would start leaking into the basolateral chamber, resulting in a total volume of 0.3 mL rather than 0.1 mL. The biomarker produced also needed to be normalised to the ATP content of each monolayer, which directly correlated with total cell number. Finally, the normalised values were compared as a percentage of the analyte produced as compared with the untreated monolayers. 

All data presented, unless specified, are expressed as mean ± standard deviation (SD) of three replicates from one representative donor. GraphPad Prism 9.0 (GraphPad software Inc., Boston, MA, USA) was used to perform the analysis. The combined data were analysed to compare the difference in toxicity elicited between VRP-034 and polymyxin B using two-way analysis of variance (ANOVA), followed by a Sidak’s multi-comparison post hoc test.

## 5. Conclusions

In conclusion, we have confirmed that the representative human donor showed uptake of FITC-albumin across the apical membrane and was partially, yet significantly, inhibited by 400 ng/mL RAP, suggesting expected functioning of the megalin/cubilin complex. With this data and combined with the responses, particularly in the biomarker production, after treatment with either polymyxin B or VRP-034, we suggest that VRP-034 is better tolerated in human PTCs than marketed polymyxin B and that this may be as a result of reduced proximal tubular uptake via the megalin/cubilin receptor complex. This suggests that this novel formulation of polymyxin B has the potential to be a better-tolerated treatment option for patients with Gram-negative bacterial infections compared to polymyxin B, and meet a substantial market demand for safer polymyxin antibiotics.

## Data Availability

The data presented in this study are available on request from the corresponding author. The data are not publicly available due to intellectual property consideration.
